# Endovascular treatment of congenital arteriovenous fistulas in the parotid region using coils and absolute ethanol

**DOI:** 10.3389/fneur.2026.1787924

**Published:** 2026-08-12

**Authors:** Zilu Wang, Yuchen Shen, Zhenfeng Wang, Yifeng Han, Lianzhou Zheng, Mingzhe Wen, Xitao Yang, Ruoyu Di, Lixin Su, Deming Wang, Xindong Fan

**Affiliations:** Department of Interventional Therapy, Multidisciplinary Team of Vascular Anomalies, Shanghai Ninth People’s Hospital, Shanghai Jiao Tong University, Shanghai, China

**Keywords:** absolute ethanol, congenital arteriovenous fistula, detachable coils, endovascular embolization, parotid gland

## Abstract

**Objective:**

To evaluate the clinical features and therapeutic outcomes of endovascular embolization for congenital arteriovenous fistulas (cAVFs) located in the parotid region.

**Methods:**

Fifteen consecutive patients with parotid cAVFs who underwent endovascular embolization between September 2017 and September 2025 were retrospectively reviewed. The cohort comprised nine males and six females, including six children (median age: 4 years) and nine adults (median age: 35 years). Clinical presentations, computed tomography imaging, and digital subtraction angiography data were analyzed. The therapeutic protocol involved primary coil embolization of the dilated draining vein to achieve flow reduction, followed by the targeted administration of absolute ethanol to obliterate the residual shunt.

**Results:**

The predominant clinical manifestations were pulsation (14/15, 93.3%), followed by thrill (7/15, 46.7%), elevated skin temperature (7/15, 46.7%), tinnitus (3/15, 20%), and hemorrhage (1/15, 6.7%). All patients were managed via combined embolization using detachable coils (median: 7), fibered coils (median: 21), and absolute ethanol (median: 10 mL). The complete response rate was 86.7% (13/15) at the 3-month and 6-month follow-ups after the initial treatment. At the 1-year follow-up, the final complete symptom resolution rate reached 100% (15/15) after repeat sessions in selected cases. Complications included transient localized swelling (15/15, 100%), temporary facial nerve paralysis (1/15, 6.7%), and late coil exposure via the external auditory canal (1/15, 6.7%), all of which resolved with targeted management.

**Conclusion:**

Endovascular embolization using a combination of coils and absolute ethanol is a clinically viable and effective strategy for the management of parotid cAVFs. While localized swelling is a universal post-procedural event, major systemic complications are rare, rendering this technique a valuable minimally invasive alternative to open surgery.

## 1 Introduction

An arteriovenous fistula (AVF) is a distinct vascular anomaly characterized by a single, direct communication between an artery and the adjacent venous system that bypasses the capillary bed (1). The etiology of AVFs typically encompasses trauma (2), infection, iatrogenesis (3), or developmental defects; the latter is clinically referred to as a congenital AVF (cAVF). Congenital AVFs originating in the parotid region are exceedingly rare, with only 10 cases documented in the current literature (4–6). Clinical manifestations of parotid cAVFs vary widely, ranging from a localized pulsatile mass and tinnitus to severe pain, headaches, and potentially life-threatening right heart overload (7).

The management of AVFs traditionally involves surgical resection, endovascular embolization, or a combined approach (8–10). However, given the complex anatomical structure of the parotid region, surgical resection carries the risk of postoperative complications, most notably facial nerve injury and Frey’s syndrome (11). Over the past few decades, endovascular embolization has emerged as a satisfactory modality for managing high-flow vascular anomalies, challenging traditional open surgery with its distinctly minimally invasive advantages (12, 13). Nevertheless, dedicated clinical research systematically evaluating the efficacy and safety of endovascular embolization specifically for parotid cAVFs remains scarce.

To address this gap in the literature, our study retrospectively analyzed a cohort of 15 consecutive patients with parotid cAVFs who were managed using a combined coil-assisted absolute ethanol embolotherapy strategy. This study aims to comprehensively evaluate the clinical outcomes, procedure-related complications, and long-term recurrence rates associated with this endovascular intervention.

## 2 Materials and methods

This retrospective review of patient medical and imaging records was approved by our Institutional Review Board, and written informed consent was obtained from all patients or their legal guardians.

### 2.1 Patient selection and eligibility criteria

**Figure 1:**
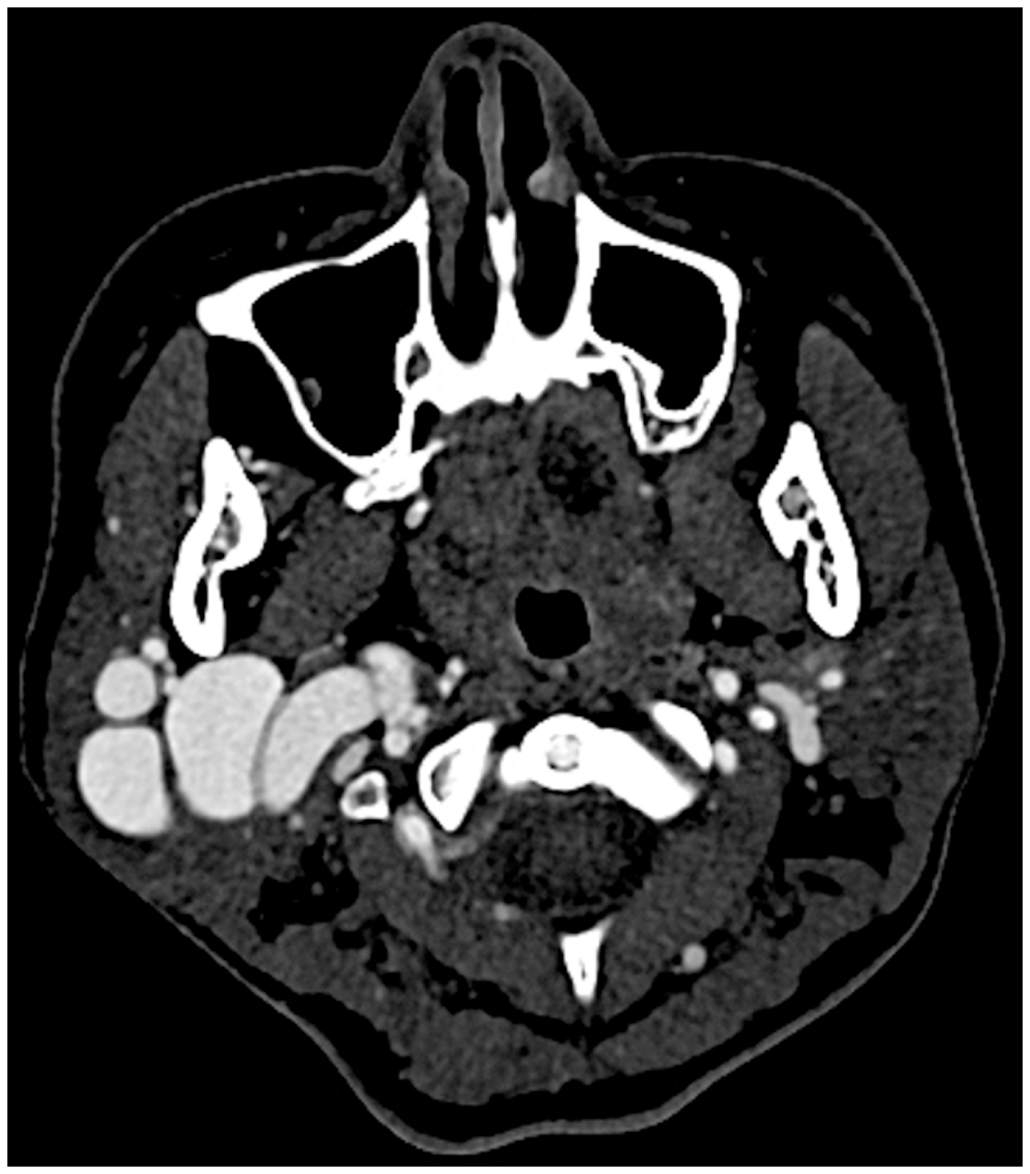
Contrast-enhanced computed tomography of a 35-year-old female with a parotid arteriovenous fistula (patient # 13). Ectatic vessels with intense enhancement were noted in the left parotid region.

**Figure 2:**
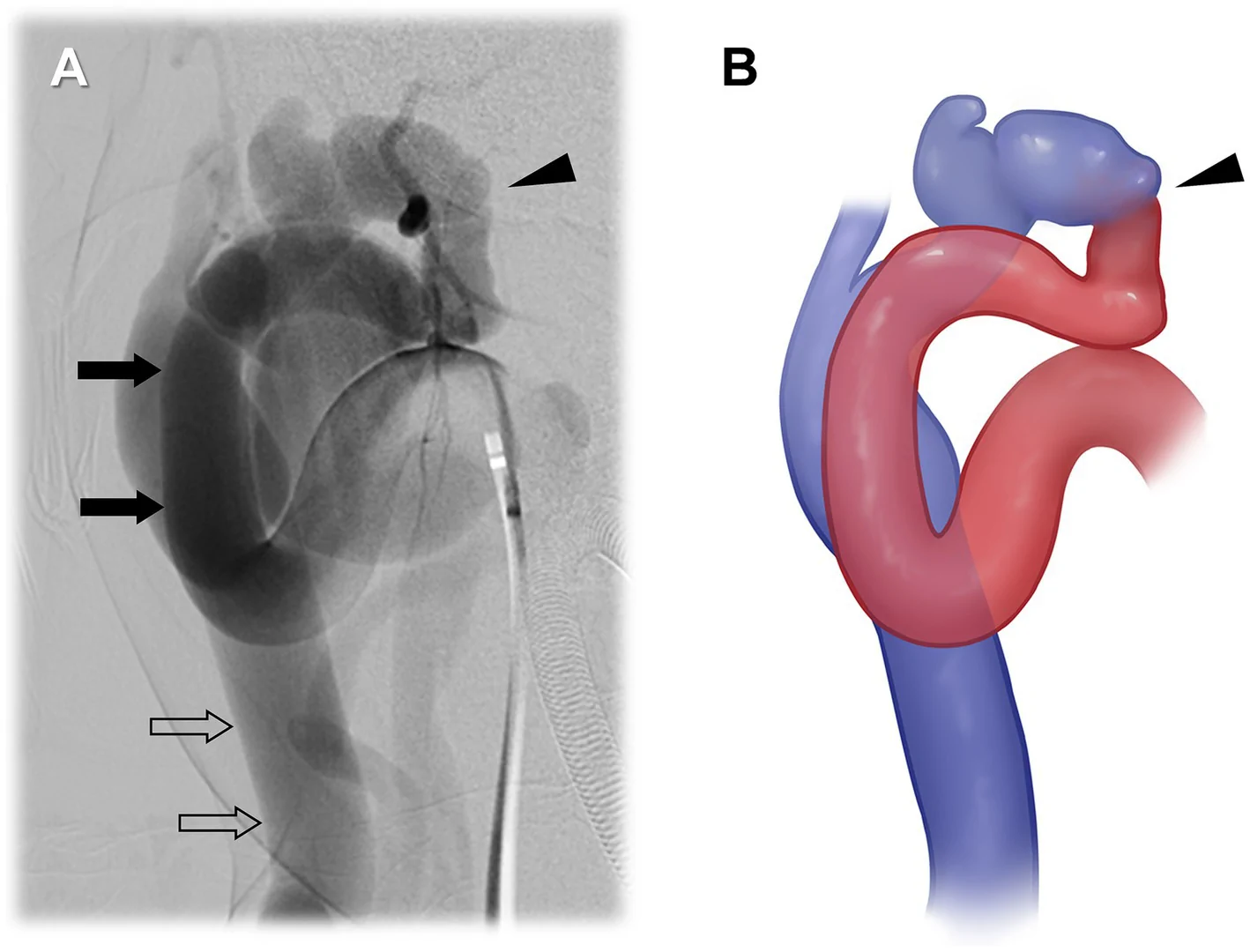
Angiographic findings of an arteriovenous fistula **(A)** and a schematic diagram depicting the location of the fistula **(B)**. The maxillary artery (black arrows) and the external jugular vein (open arrows) were abnormally enlarged on angiography **(A)**. The fistula was located in between as a distinct stenosis (A, B, arrowheads), and was identified as the ideal embolization site.

Between September 2017 and September 2025, 15 consecutive patients diagnosed with parotid cAVFs who underwent endovascular embolization at our institution were enrolled in this study. The diagnosis was initially established using preprocedural contrast-enhanced computed tomography (CT; Figure 1) and subsequently confirmed by digital subtraction angiography (DSA; Figure 2A). Patients were excluded if they presented with a history of trauma, infection, or prior regional surgery, or if they had suspected arteriovenous malformations (AVMs). A comprehensive retrospective analysis of the patients’ clinical and radiological data was performed, and their baseline characteristics are summarized in Table 1.

**Table 1:** Baseline clinical data of patients with parotid cAVF.

Patient number	Sex^*^	Age (years)	Lesion side	Feeding artery^†^	Draining vein(s)^‡^	Clinical manifestations^§^	Duration of manifestations
1	M	20	Right	FA	IJV	EST, pulsation, thrill	3 years
2	M	3	Left	MA	IJV, EJV	EST, pulsation, thrill	3 years
3	M	33	Right	MA	EJV	EST, pulsation, thrill, tinnitus	3 years
4	M	4	Right	STA	EJV	Pulsation, thrill	4 years
5	M	53	Right	MA	IJV	EST, pulsation	5 months
6	F	30	Left	MA	EJV	Thrill, tinnitus	3 years
7	M	39	Right	MA	IJV, EJV	Pulsation, tinnitus, hemorrhage	30 years
8	F	3	Right	MA	IJV, EJV	EST, pulsation	2 years
9	F	4	Right	MA	IJV, EJV	EST, pulsation, thrill	2 months
10	F	32	Left	MA	IJV, EJV	Pulsation	3 months
11	F	4	Right	MA	IJV, EJV	Pulsation	1 year
12	M	38	Left	MA	EJV	EST, pulsation, thrill	38 years
13	F	35	Right	MA	EJV	Pulsation	35 years
14	M	45	Right	STA	EJV	Pulsation	10 months
15	M	8	Right	ECA	IJV, EJV	Pulsation	2 months

*F, female; M, male. ^†^ FA, facial artery; MA, maxillary artery; STA, superficial temporal artery; ECA, external carotid artery. ^‡^ IJV, internal jugular vein; EJV, external jugular vein. ^§^ EST, elevation of skin temperature.

### 2.2 Procedural details of embolotherapy

#### 2.2.1 Preprocedural preparation and vascular access

**Figure 3:**
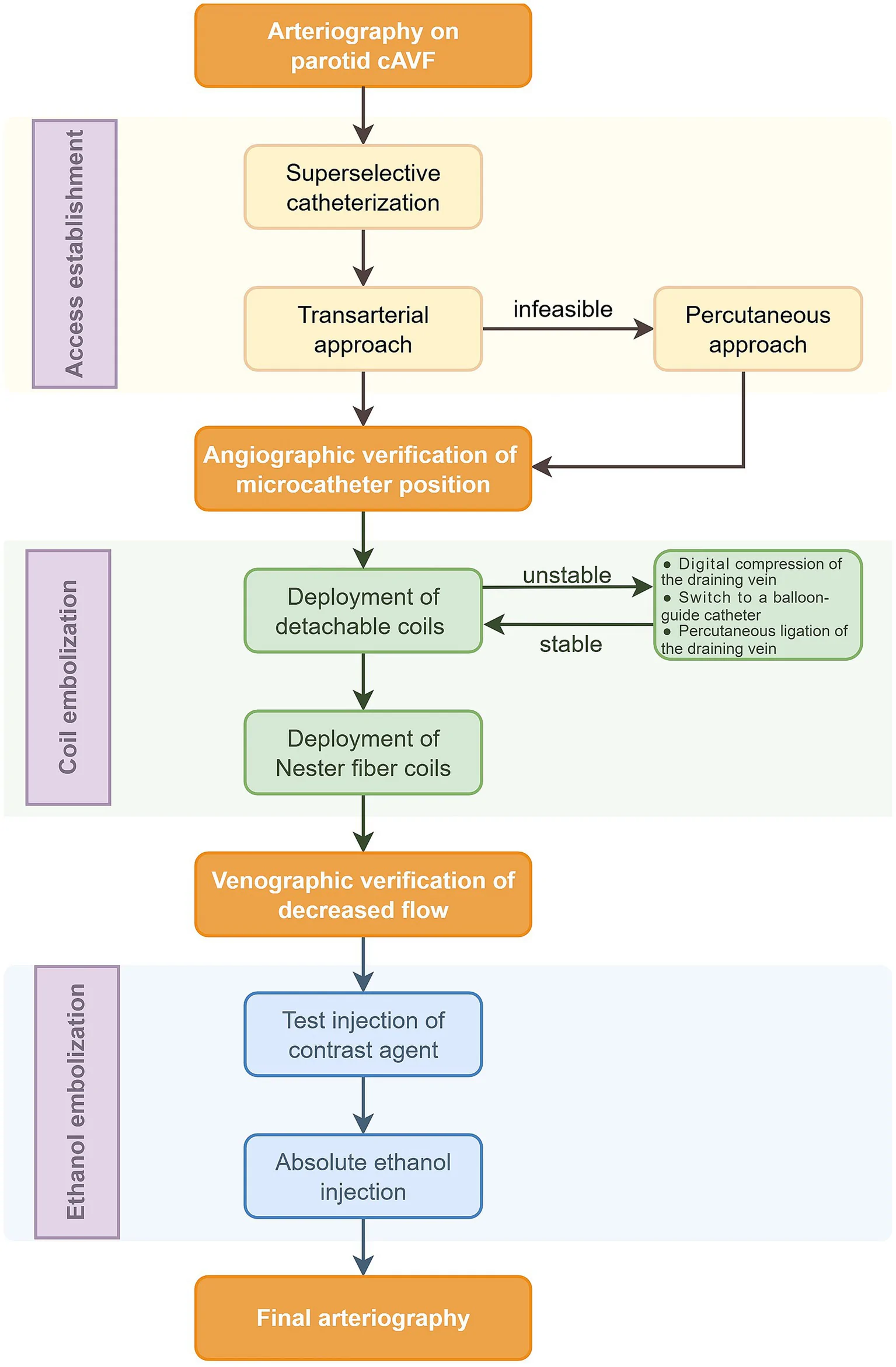
Schematic diagram of the combined embolization technique with coils and ethanol. cAVF, congenital arteriovenous fistula.

All endovascular procedures were performed under general anesthesia, with continuous arterial pressure monitoring via a radial arterial catheter. Embolization was executed by two experienced interventional radiologists using either a transarterial or a percutaneous approach; the transarterial route was prioritized, while the percutaneous direct-puncture route served as a backup option (Figure 3).

Following initial femoral arterial access, baseline selective angiography of the internal and external carotid arteries was performed. For the transarterial approach, a 2.4F microcatheter (Boston Scientific Corporation, MA, USA) was advanced coaxially through a 5F guiding catheter into the target feeding artery to reach the draining vein. In cases where severe arterial tortuosity precluded transarterial navigation, a percutaneous approach was utilized: the dilated venous sac was directly punctured using an 18-G needle (Cook, Bloomington, IN, USA), through which the microcatheter was introduced. In all patients, the microcatheter tip was precisely positioned within the venous sac immediately distal to the fistula (Figure 2B), as confirmed by selective angiography.

#### 2.2.2 Coil deployment and flow-reduction techniques

**Figure 4:**
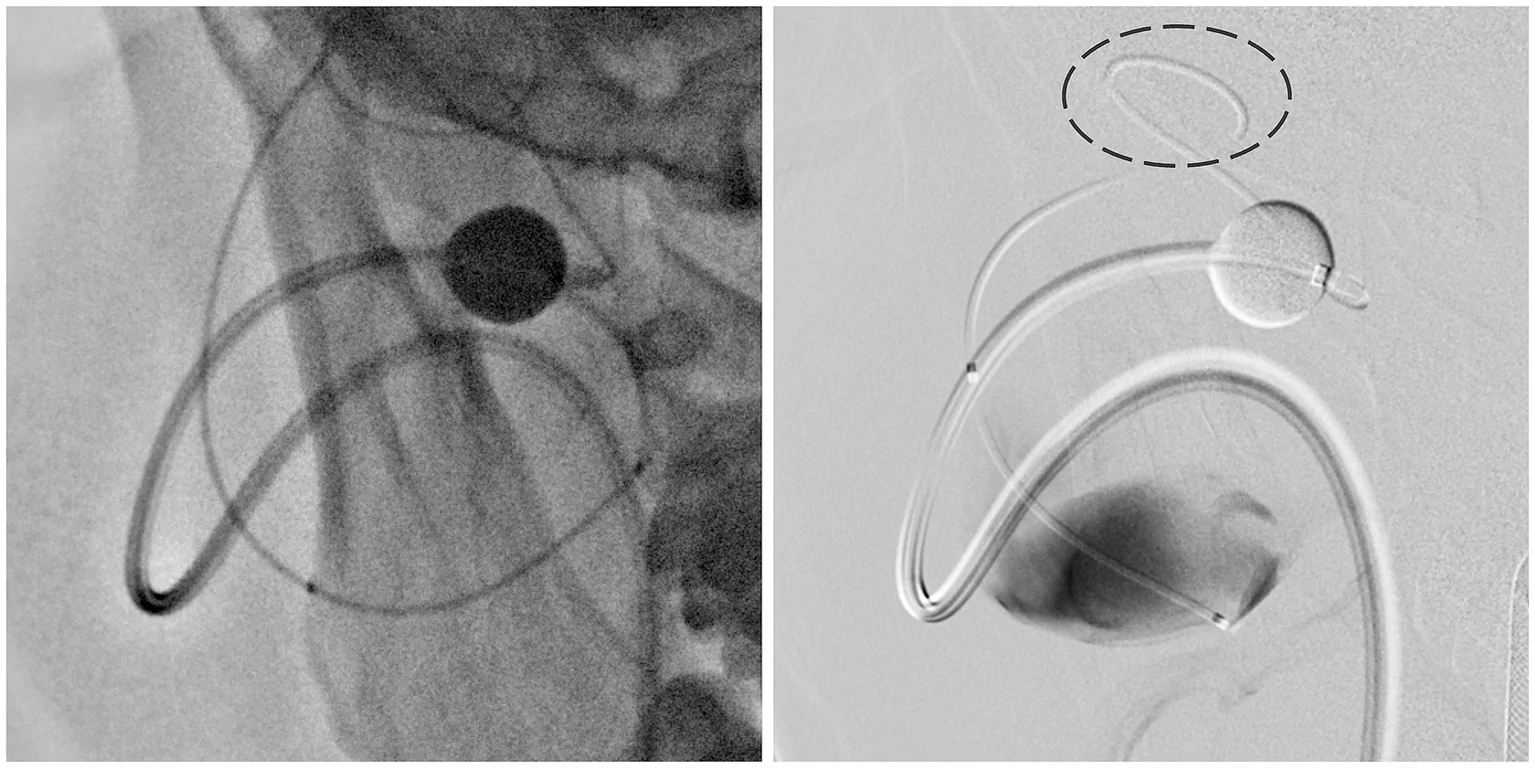
Balloon-assisted embolization of a high-flow parotid arteriovenous fistula. The balloon guide catheter was positioned in the feeding artery proximal to the fistula. A microcatheter was advanced coaxially through the guide catheter, passed through the winding fistula (dashed outline), and positioned within the venous sac. After confirmation of the position by venography, the balloon was inflated with contrast, thereby arresting the arterial inflow (**A**, fluoroscopy; **B**, subtracted angiography). This auxiliary technique is suitable for high-flow arteriovenous fistulas and effectively reduces the risk of coil migration.

**Figure 5:**
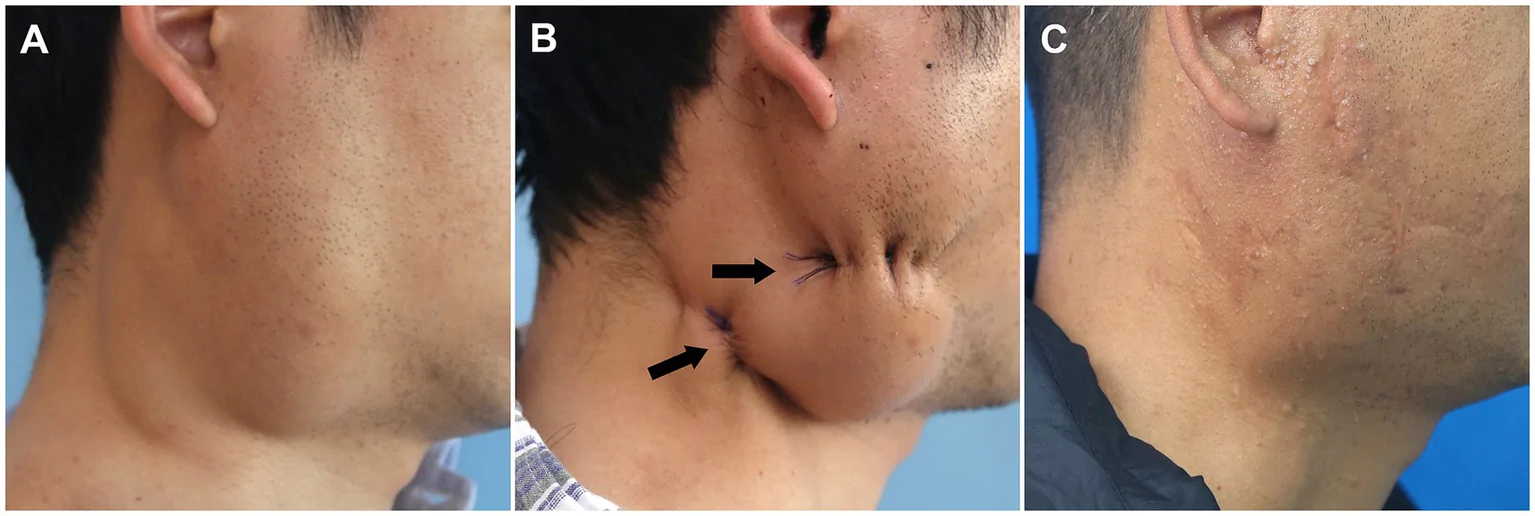
Adjunctive percutaneous venous ligation to stabilize coil deployment in a high-flow parotid arteriovenous fistula. **(A)** A 33-year-old male with a parotid arteriovenous fistula presented with a markedly dilated, prominent vein over the right parotid region. **(B)** The draining vein was ligated percutaneously using 3–0 absorbable suture (arrows) before coil detachment to stabilize the rapid hemodynamics and facilitate coil configuration. **(C)** The patient’s appearance recovered well at 6-year follow-up, leaving only an inconspicuous scar at the ligation site.

Detachable coils (Boston Scientific Corporation, MA, USA) were introduced through the microcatheter and deployed under continuous fluoroscopic guidance. Coils were retracted and redeployed if an unfavorable position or configuration was observed. When high-flow hemodynamics prevented the achievement of a stable coil frame, one or more of the following auxiliary flow-reduction techniques were implemented to stabilize the rapid blood flow and facilitate coil configuration:

- Manual digital compression of the draining vein;
- Deployment of a balloon guide catheter (Ricoton, China), with the balloon inflated with contrast medium to achieve temporary flow arrest (Figure 4);
- Percutaneous ligation of the superficial draining vein using a 3–0 absorbable suture (Figure 5).

Following the establishment of a stable detachable coil configuration within the draining vein, Nester fiber coils (Cook, Bloomington, IN, USA) were consecutively packed as densely as possible to achieve maximal mechanical embolization.

#### 2.2.3 Ethanol sclerotherapy and endpoint evaluation

**Figure 6:**
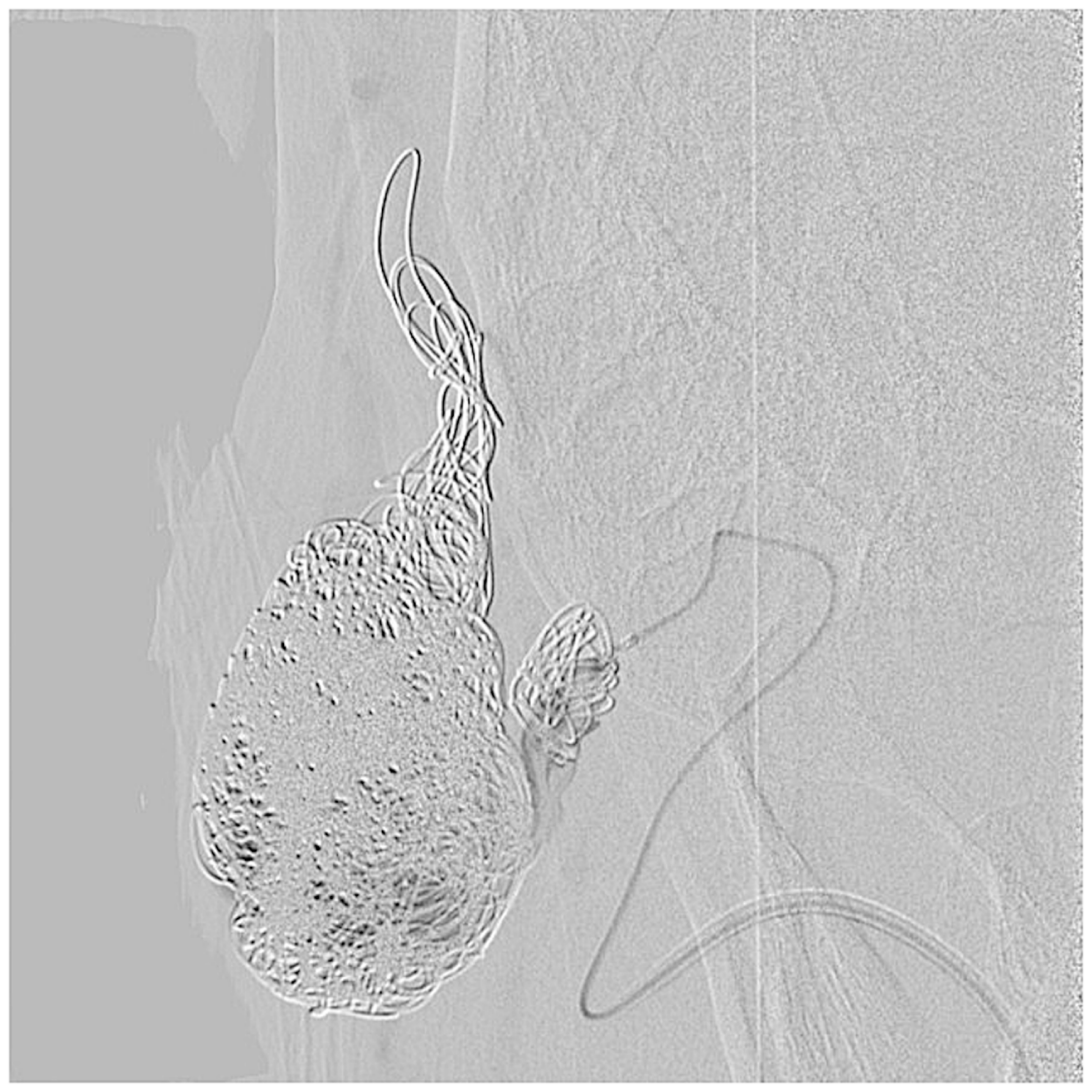
Ethanol embolization after coil placement. Following a substantial flow reduction by coil packing, absolute ethanol was injected at the fistula to achieve definitive occlusion.

Prior to ethanol embolization, angiography was performed to verify a significant reduction in blood flow achieved by the densest possible coil packing (Figure 6). To determine the precise volume of sclerosant required and minimize the risk of systemic migration, a test injection of contrast agent was performed under fluoroscopy. The required volume of absolute ethanol was calibrated to match the exact amount of contrast agent needed to completely fill the residual vascular space of the lesion. Absolute ethanol (99.9%, China National Medicines Guorui Pharmaceutical Company Limited, China) was then manually injected via the microcatheter to obliterate the residual cAVF lesions. Finally, a completion arteriography was performed 10 min post-injection to verify complete occlusion of the fistula.

### 2.3 Follow-up strategy and clinical evaluation

Patients were systematically evaluated via clinical examination and Doppler ultrasonography (DUS) at 3, 6, and 12 months post-procedurally, and annually thereafter. DSA was recommended at the 12-month follow-up interval to evaluate anatomical outcomes. During routine subsequent follow-ups, angiography was not routinely performed unless clinical symptoms or DUS findings raised suspicion of lesion recurrence.

Therapeutic outcomes were categorized into three tiers based on a combination of clinical presentations and radiological findings:

- Complete Response: Full resolution of clinical symptoms and the complete absence of arteriovenous shunting on DUS.
- Partial Response: Persistence of residual clinical symptoms, or a greater than 50% reduction/disappearance of the lesion size demonstrated on follow-up angiograms.
- No Response: Absence of symptomatic relief, documented clinical deterioration, or a less than 50% reduction/disappearance of the lesion size on follow-up angiograms.

## 3 Results

### 3.1 Patient demographics and clinical characteristics

This study involved a total of 15 patients (9 males, 6 females). The cohort comprised six children with a median age of 4 years (interquartile range [IQR]: 3.25–4 years) and nine adults with a median age of 35 years (IQR: 32–39 years). Lesions were located on the right side in 11 of 15 patients (73.3%). The predominant clinical manifestation was pulsation (14/15, 93.3%), followed by thrill (7/15, 46.7%), elevated skin temperature (7/15, 46.7%), tinnitus (3/15, 20%), and hemorrhage (1/15, 6.7%; Table 1). Symptoms had been present since birth in 4 patients (26.7%).

### 3.2 Angiographic findings and angioarchitecture

Super-selective angiography demonstrated that the ipsilateral maxillary artery was the most frequent feeding artery of the cAVFs in 11 of 15 cases (73.3%), followed by the superficial temporal artery (2/15, 13.3%) and the facial artery (1/15, 6.7%), and the external carotid artery (1/15, 6.7%). Venous drainage of the parotid cAVFs occurred via the external jugular vein (6/15, 40%), the internal jugular vein (2/15, 13.3%), or a combination of both (7/15, 46.7%; Table 1).

### 3.3 Embolization modalities

Regarding the vascular access strategy, the intervention route was transarterial in 7 patients (46.7%), percutaneous in 3 (20%), and a combined approach in the remaining 5 (33.3%). Embolization was performed using detachable coils (median: 7, IQR: 4–14.5), fibered coils (median: 21, IQR: 5–60.5), and absolute ethanol (median: 10 mL, IQR: 4.5–16.5 mL; Table 2).

**Table 2:** Treatment details of initial sessions and follow-up results.

Patient number	Endovascular access	Auxiliary flow-reduction techniques	Embolization materials			Postprocedural symptoms & complications	Evaluation				Follow-up duration (month)
			Detachable coils	Fiber coils	Ethanol (mL)		3-month follow-up	6-month follow-up	12-month follow-up	Latest follow-up	
1	Transarterial		3	0	3	Swelling	CR	CR	CR	CR	84
2	Transarterial, percutaneous		4	20	14	Swelling	PR	CR	CR	CR	85
3	Transarterial	Percutaneous ligation	2	65	9	Swelling	CR	CR	CR	CR	82
4	Transarterial		6	20	3	Swelling	CR	CR	CR	CR	81
5	Percutaneous		7	48	14	Swelling	CR	CR	CR	CR	80
6	Transarterial		2	21	5	Swelling	CR	CR	CR	CR	94
7	Transarterial, percutaneous		8	146	33	Swelling, facial nerve paralysis, coil exposure	CR	PR	CR	CR	77
8	Transarterial percutaneous		5	0	10	Swelling	CR	CR	CR	CR	44
9	Transarterial		4	10	2	Swelling	CR	CR	CR	CR	47
10	Transarterial	Digital compression	14	82	16	Swelling	CR	CR	CR	CR	44
11	Transarterial percutaneous		12	0	4	Swelling	CR	PR	CR	CR	35
12	Transarterial percutaneous	Digital compression	46	99	25	Swelling	PR	CR	CR	CR	38
13	Transarterial	Balloon guide catheter	26	56	10	Swelling	CR	CR	CR	CR	23
14	Percutaneous		21	40	25	Swelling	CR	CR	CR	CR	17
15	Percutaneous		15	0	17	Swelling	CR	CR	CR	CR	3
	CR rate						86.7%	86.7%	100%	100%	

CR, complete response, defined as resolution of clinical symptoms and absence of arteriovenous shunting on Doppler ultrasonography; PR, partial response, defined as residual symptoms or disappearance of more than half of the lesion on angiograms. n.a., not applicable.

### 3.4 Clinical outcomes

**Figure 7:**
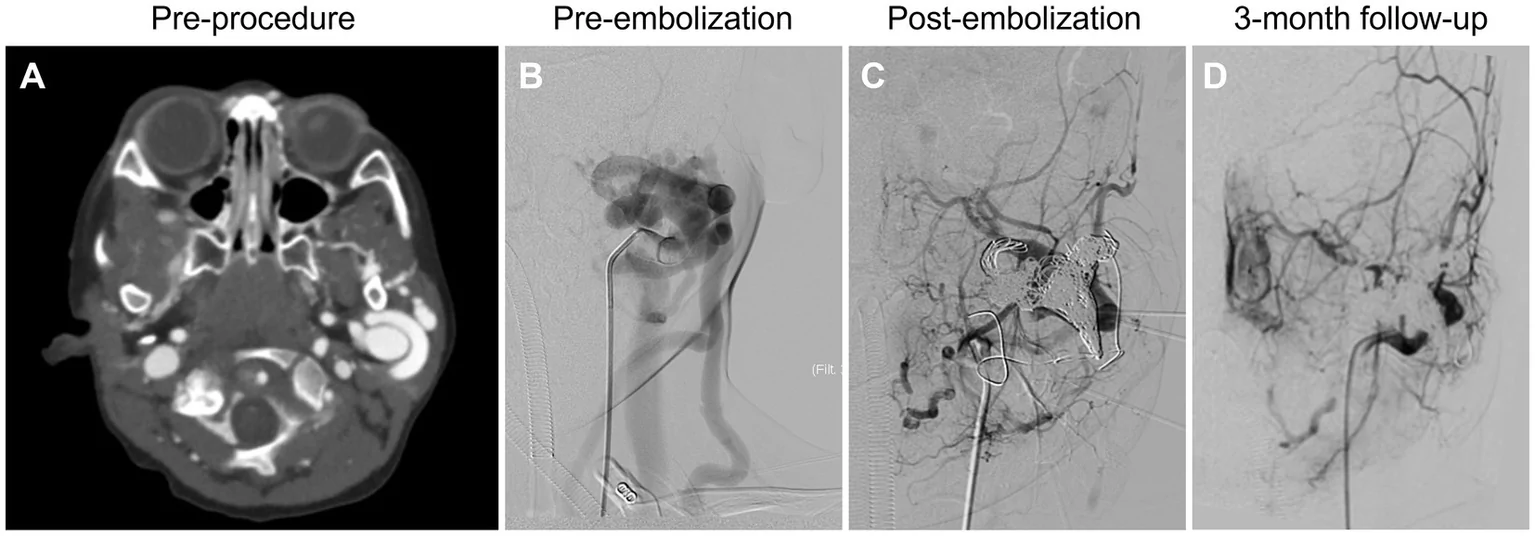
Endovascular embolization of a left parotid arteriovenous fistula in a 3-year-old boy (Patient # 2). **(A)** Preprocedural contrast-enhanced computed tomography revealed dilated, enhanced vessels in the left parotid region. **(B)** Digital subtraction angiography confirmed the fistula with a tortuous feeding artery and early venous opacification. **(C)** Post-embolization angiography showed complete occlusion of the fistula with opacification of distal arterial branches, indicating improved distal perfusion. **(D)** Angiography at 3-month follow-up demonstrated no recurrence of the fistula.

**Figure 8:**
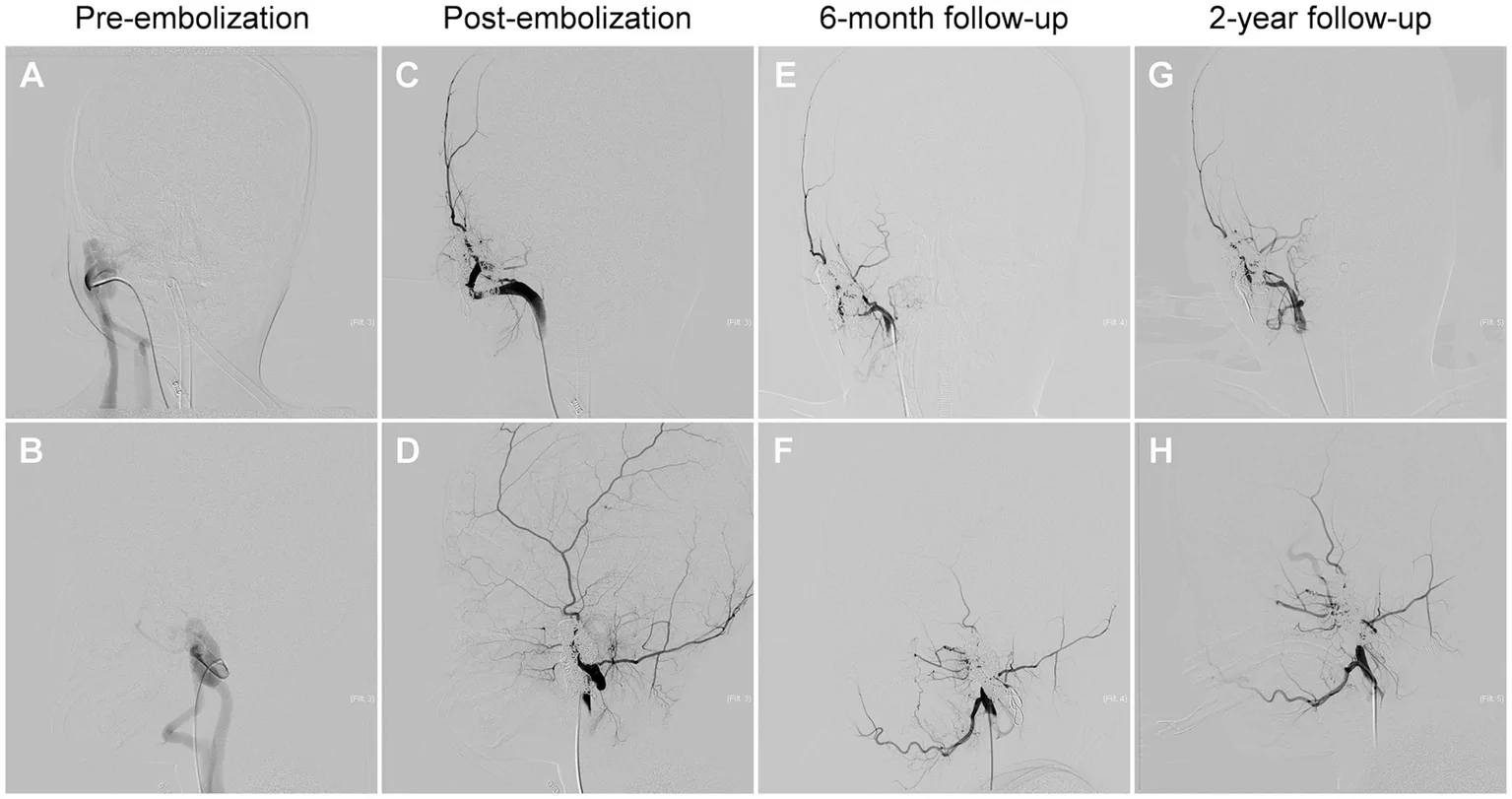
Endovascular embolization of a right parotid arteriovenous fistula in a 4-year-old girl (Patient # 11). Pre-embolization anteroposterior **(A)** and lateral **(B)** digital subtraction angiography identified a fistula supplied by the contralateral maxillary artery and draining into the external and internal jugular veins. Post-embolization angiography **(C,D)** confirmed complete occlusion of the fistula and opacification of distal arterial branches. Angiography after 6 months **(E,F)** and 2 years **(G,H)** showed sustained occlusion without evidence of recanalization.

The follow-up duration ranged from 3 to 94 months, with a median of 47 months. The complete response rates at 3, 6, and 12 months after the initial procedure were 86.7% (13/15), 86.7% (13/15), and 100% (15/15), respectively (Table 2; Figures 7, 8).

Specifically, Patient # 2 and # 12 presented with residual slight pulsation at the 3-month follow-up. Subsequent angiography revealed complete obliteration of the fistula in Patient # 2 (with the pulsation resolving spontaneously thereafter), whereas Patient # 12 demonstrated recanalization and underwent a second session of 5 mL of ethanol embolization. At the 6-month follow-up, a recurrence of pulsation occurred in Patients # 7 and # 11, both of whom achieved cure after repeat ethanol embolotherapy (1 mL and 3 mL, respectively). By the 12-month follow-up, all patients had achieved complete symptom resolution, resulting in an overall complete response rate of 100% (15/15).

### 3.5 Complications

No major systemic adverse events, such as cardiopulmonary accidents, transient ischemic attacks, or strokes, were reported. Transient localized swelling occurred in all patients (15/15, 100%) following absolute ethanol embolization; however, skin or mucosal necrosis was not observed. Patient # 7 experienced temporary facial nerve paralysis following embolization of the feeding maxillary artery (Table 2). This can be attributed to mechanical nerve compression caused by adjacent tissue edema following ethanol irritation, which completely resolved following a three-day course of intravenous dexamethasone (10 mg daily). Six months after the procedure, the same patient presented with delayed coil exposure at the external auditory canal, which corresponded to the site of the original hemorrhage. The exposed coils were successfully managed via local debridement without further sequelae.

## 4 Discussion

Arteriovenous fistulas (AVFs) are vascular lesions characterized by a single, direct communication between an artery and a vein. In general, AVFs are secondary to definable etiologies, including trauma (7), iatrogenesis (3), or connective tissue disease (14); whereas congenital or spontaneous AVFs remain exceedingly rare. The persistence of embryonic communications during embryonic vascular development is widely considered the pathophysiological basis of cAVFs (15). In the head and neck region, cAVFs are most commonly encountered in the cervical area as carotid-jugular fistula (16–18), followed by the parotid region. A review of the literature revealed that only 10 cases of parotid cAVFs have been previously documented (4–6) (Table 3). In the present study, we evaluated a cohort of 15 patients with parotid cAVFs, all of whom were successfully managed via coil-assisted absolute ethanol embolotherapy and achieved complete clinical resolution during long-term follow-up.

**Table 3:** Summary of previously reported cases and treatment strategies for parotid congenital arteriovenous fistulas.

Authors	Sex	Age (years)	Symptoms	Treatment	Embolization materials	Result	Follow-up duration (years)
Gobin et al. (5)	M	12	Pulsatile mass, bruit	Endovascular	Balloon	Cure	12
	M	3	Pulsatile mass	Endovascular	Balloon	Cure	6
	M	5	Pulsatile mass	Endovascular	Balloon	Cure	5
	F	4	Pulsatile mass	Endovascular	Balloon	Cure	4
	F	2	Pulsatile mass	Endovascular	Balloon	Cure	7
	F	56	Pulsatile mass, bruit	Endovascular	Balloon and NBCA	Cure	5
Gabrielsen et al. (4)	M	45	Pulsatile mass, tinnitus	Endovascular	Balloon	Cure	6 months
	F	34	Pulsatile mass, tinnitus	Endovascular	Balloon	Cure	9 months
	M	5 months	Pulsatile mass, bruit	Surgery	/	Cure	3
Horiuchi et al. (6)	M	27	Diminished consciousness; ataxic gait, pulsatile mass, bruit	Endovascular	Coils	Cure	Not mentioned

The clinical manifestations of AVFs stem directly from the abnormal hemodynamics induced by the arteriovenous shunt. Common signs and symptoms include a pulsatile mass, bruits, thrills, and localized swelling with associated cosmetic deformity. In severe cases, patients may present with localized pain, acute hemorrhage, or even progress to congestive heart failure. Pulsatile tinnitus is a hallmark symptom of parotid cAVFs due to their intimate anatomic proximity to the auditory apparatus. Within our cohort, pulsation (93.3%), thrill (46.7%), and elevated local skin temperature (46.7%) were the primary complaints. Tinnitus was documented in 3 patients (20%). Notably, one adult patient presented with acute, severe preprocedural hemorrhage, whereas no cases of cardiac failure, parotid gland dysfunction, or facial nerve impairment were observed.

Clinically, AVFs can be identified by a palpable thrill, an auscultated bruit over the lesion, or a positive Branham-Nicoladoni sign (7). However, imaging remains indispensable for diagnostic confirmation and precise localization of the fistula. The imaging modalities for AVFs include Doppler ultrasonography (DUS), computed tomography (CT), magnetic resonance imaging (MRI), and digital subtraction angiography (DSA). DUS serves as an essential, non-invasive screening modality that is highly effective for evaluating real-time shunt hemodynamics (19). While CT and MRI each possess unique strengths in delineating spatial relationships with adjacent anatomical structures, DSA provides the most comprehensive information regarding angioarchitecture and flow dynamics, remaining the gold standard for AVF diagnosis (20). In this study, all patients underwent preprocedural contrast-enhanced CT for initial anatomical evaluation. However, due to severe metallic artifacts generated by packed coils on post-procedural CT, DUS was selected as the primary imaging modality for routine follow-up. In contrast, invasive DSA was reserved exclusively for concurrent endovascular re-interventions or cases where lesion recurrence was strongly suspected based on clinical or ultrasonographic deterioration.

The primary therapeutic goal for an AVF is to achieve complete elimination of the fistula. This endpoint can be accomplished via either surgical excision or endovascular embolization. For cAVFs located in the parotid region, the vascular lesions are commonly embedded deep within the parotid gland tissue. Consequently, intraoperative exposure of the fistula inevitably hazards critical anatomical structures, most notably the parotid parenchyma and the branches of the facial nerve. Moreover, if the integrity of the cAVF is accidentally compromised during surgical dissection, profuse arterial hemorrhage can occur, significantly compromising the surgical field and increasing procedural difficulty. Therefore, endovascular therapy is highly recommended owing to its minimally invasive profile. The technical crux of endovascular embolization is the precise localization of the fistula, which is typically characterized by a sudden narrowing between the feeding artery and the ectatic draining vein (Figure 2B). Sole ligation or embolization of the feeding arteries, regardless of the embolic agent used, leads to a high rate of cAVF recurrence due to collateral recruitment (21).

The type of embolization material in AVFs treatment varies depending on the diameter and blood velocity of the fistula. Options include particulate embolic agents, liquid embolic agents, as well as detachable balloons, coils, and covered stents. In parotid cAVFs, the rapid blood flow at the fistula leads to high risks of ectopic embolization when delivering particulate embolic agents or liquid embolic agents (22). Stents were considered when occlusion of the supplying artery may lead to serious complications, but they are not suitable for tortuous vessels (18). Detachable balloons were the mainstream embolization material in the treatment of parotid cAVFs and demonstrated satisfactory fistula obliteration (4, 5) (Table 3). Relapse occurred in one case, which might be attributed to a delayed migration of the balloon caused by premature deflation or repeated movement of the mandible (15). Coils are widely utilized due to excellent mechanical stability and controlled deployment. However, using coils alone carries a risk of incomplete occlusion or long-term recanalization due to the lack of permanent endothelial destruction (23). To achieve definitive closure, liquid embolic agents are frequently introduced. Cyanoacrylates, such as N-butyl-2-cyanoacrylate (NBCA), polymerize rapidly upon contact with blood and offer fast occlusion. Yet, NBCA requires precise delivery timing, as its fast, non-cohesive polymerization poses a high risk of catheter entrapment or unpredictable distal migration in high-flow regions (24). In contrast, ethylene vinyl alcohol copolymer (EVOH) precipitates slowly from an organic solvent, thus allowing for prolonged, highly controlled injections and excellent deep penetration. However, EVOH delivery requires the injection of dimethyl sulfoxide (DMSO), which can induce severe localized pain and vasospasm (25). Its routine application specifically for parotid cAVFs remains constrained by limited clinical literature.

Absolute ethanol represents a fundamentally different therapeutic mechanism. It is a highly potent sclerosant that induces immediate protein denaturation, endothelial cell destruction, and subsequent transmural thrombosis, effectively eliminating the endothelial triggers of relapse. Absolute ethanol was selected in this cohort based on its well-established safety and efficacy in the treatment of high-flow vascular malformations, particularly arteriovenous malformations (26–28). Furthermore, our medical center has over a decade of extensive experience with ethanol embolotherapy. However, the use of absolute ethanol also carries a well-documented and severe toxicity profile (29, 30). Locally, its intense tissue irritation can cause massive swelling or skin necrosis. Systemically, rapid influx of ethanol into the central circulation can trigger catastrophic cardiopulmonary complications, including pulmonary hypertension, right ventricular failure, and cardiac arrest. To safely harness this therapeutic potency while mitigating its toxicity, patients with parotid cAVFs were treated with coil-assisted ethanol embolization (Figure 3). The decreased blood flow after coil embolization prolonged the contact time of ethanol with the fistula wall, allowing a lower, safer volume of ethanol to achieve efficient, localized endothelial ablation while minimizing the risk of systemic circulation and non-target migration. Follow-up results further verified the safety and effectiveness of this technique. Ectopic embolization caused by coil migration or cardiopulmonary accidents due to ethanol administration were not observed. Follow-ups at 12 months after the initial procedure revealed no recurrence of fistula.

Transarterial access was initially attempted as the preferred routing strategy because it provided more straightforward anatomical access to the fistula and facilitated more precise, controlled coil deployment. Despite the inherent risks of localized hematoma and infection, direct percutaneous puncture offered a highly feasible alternative access route for cases involving severely tortuous feeding arteries. In our series, 7 patients were managed entirely via a transarterial approach, whereas 3 cases required exclusively direct percutaneous puncture access. The remaining 5 patients underwent a combined approach involving transarterial coil deployment followed by percutaneous ethanol injection. This hybrid strategy was specifically adopted when the post-coiling microcatheter position remained too proximal on the arterial side; in such scenarios, *in situ* ethanol injection would carry a high risk of non-target distal embolization. To deliver the sclerosant safely, a secondary percutaneous access was established directly into the venous sac, allowing for isolated and controlled absolute ethanol administration.

The procedure-related complications observed in our cohort were generally transient and clinically manageable. Post-procedural localized swelling represents a well-documented, self-limiting inflammatory response secondary to ethanol-induced endothelial ablation, which was universally observed (100%) in our patients. Although the acute preprocedural hemorrhage in Patient # 7 was successfully controlled following emergency embolization, this individual subsequently developed temporary facial nerve paralysis and delayed coil exposure. Post-procedural facial nerve impairment in this anatomical region can be attributed to mechanical nerve compression caused by adjacent tissue edema following ethanol irritation, which completely resolved following a three-day course of intravenous dexamethasone. At the six-month follow-up, the coils exposed at the external auditory canal, which was the initial site of hemorrhage, were successfully managed via local debridement.

Furthermore, the embolic materials in this study have their inherent limitations. Coils possess long-term limitations as permanent metallic implants, including foreign-body tissue reactions and the generation of metallic artifacts on follow-up imaging. Additionally, cosmetic deformities resulting from the mass effect of densely packed coils may arise in patients presenting with large-volume fistulas. For this specific subgroup, a secondary plastic surgery could be considered as a viable management option, provided that durable, complete fistula obliteration has been radiologically confirmed after a minimum follow-up period of 2 years. Currently, ethanol embolotherapy lacks universally standardized, objective dosing guidelines, and the procedure relies substantially on the experience of the operator. This limitation is partly due to the radiolucency of absolute ethanol, which presents a major technical challenge in real-time monitoring of the injection endpoint. In the future, the development of radiopaque ethanol is expected to enhance procedural safety and facilitate broader clinical application of this technique (31).

Several limitations of this study must be acknowledged. First, its retrospective, single-center design inherently introduces potential selection and information biases. Second, the sample size is relatively small (*n* = 15), a constraint directly tied to the extreme rarity of parotid cAVFs. Third, there was a lack of a concurrent control or comparison group, making it difficult to directly measure the superiority of one technique over another. Lastly, angiographic follow-up was limited, as long-term evaluation relied predominantly on clinical symptom resolution and DUS rather than routine post-operative imaging for stable patients. Consequently, statements regarding definitive safety profiles and treatment guidelines must be interpreted with caution. Considering its distinct minimally invasive advantages, endovascular therapy represents a highly viable alternative to surgery for parotid cAVFs, though prospective, multi-center studies with rigorous comparative cohorts are warranted to establish formal clinical recommendations.

## 5 Conclusion

Congenital AVFs originating in the parotid region represent an exceedingly rare subset of vascular anomalies. To the best of our knowledge, this study evaluates a cohort of 15 patients, constituting the largest series focused on parotid cAVFs reported in a single study to date. Our institutional data indicate that the combined endovascular technique utilizing both coils and absolute ethanol is a clinically feasible, effective, and manageable strategy for this rare entity, yielding favorable clinical outcomes over a median follow-up duration of 47 months. Further prospective, multi-center investigations with larger sample sizes are warranted to definitively establish long-term safety profiles and standardized treatment recommendations.

## Data Availability

The raw data supporting the conclusions of this article will be made available by the authors, without undue reservation.
